# Ectopic Liver Tissue in the Gall Bladder: A Rare Entity

**DOI:** 10.7759/cureus.6323

**Published:** 2019-12-08

**Authors:** Suman Baral, Shrinit Babel, Neeraj Thapa, Raj K Chhetri

**Affiliations:** 1 Surgery, Lumbini Medical College and Teaching Hospital, Tansen, NPL; 2 Miscellaneous, Steinbrenner High School, Florida, USA

**Keywords:** ectopic liver tissue, hepatocellular carcinoma

## Abstract

Ectopic liver tissue (ELT) is a rare clinical entity that any surgeon faces in their career. Due to the association or propensity to develop hepatocellular carcinoma, this disease has gained clinical importance, and surgeons ought to be aware of the possible intervention and complications that can be associated with it. Incidence has been reported to be 0.24%-0.47%, with the gall bladder being the most common site. Anatomically, ELT in the gall bladder derives its blood supply either from the vascular pedicle arising with or without its own vein from the liver parenchyma or from branches of the cystic artery and, sometimes, through vascular structures embedded within the mesentery lying adjacent to the liver parenchyma. Surgically, it becomes important to delineate the blood supply because, often, the operating surgeon might encounter uncontrollable bleeding if the blood supply has been derived from the liver parenchyma itself. Complications that can be associated with ectopic liver are torsion, bleeding into the peritoneum, cirrhosis, and, sometimes, lead to malignant degeneration to hepatocellular carcinoma. It can be due to metabolic inactivity owing to less efficient vascular and biliary ductal systems, which sometimes might be confused for occult metastases from a primary hepatoma. Gall bladder-associated ELT is best managed by en bloc resection via laparoscopic cholecystectomy, which suffices if the biopsy comes out to be negative. However, as the risk of malignant degeneration still exists in about 3% of cases, some patients might need to undergo a second surgery for a negative resection margin and regional lymphadenectomy.

## Introduction

Ectopic liver tissue (ELT) is a rare clinical entity due to an error of morphogenesis in which the growth of normal liver tissue occurs outside its normal anatomic location [1]. Any surgeon can encounter ELT in their surgical career as a clinical or radiological challenge, sometimes mimicking a fascinoma, or during laparoscopy or open operations. Additionally, due to the association or propensity to develop hepatocellular carcinoma, ELT recognition should gain clinical importance and surgeons need to be aware of the possible variation [2]. In order to highlight its importance, this paper objectifies a rare clinical scenario that we encountered in our practice and highlights the possible management strategies.

## Case presentation

**Figure 1:**
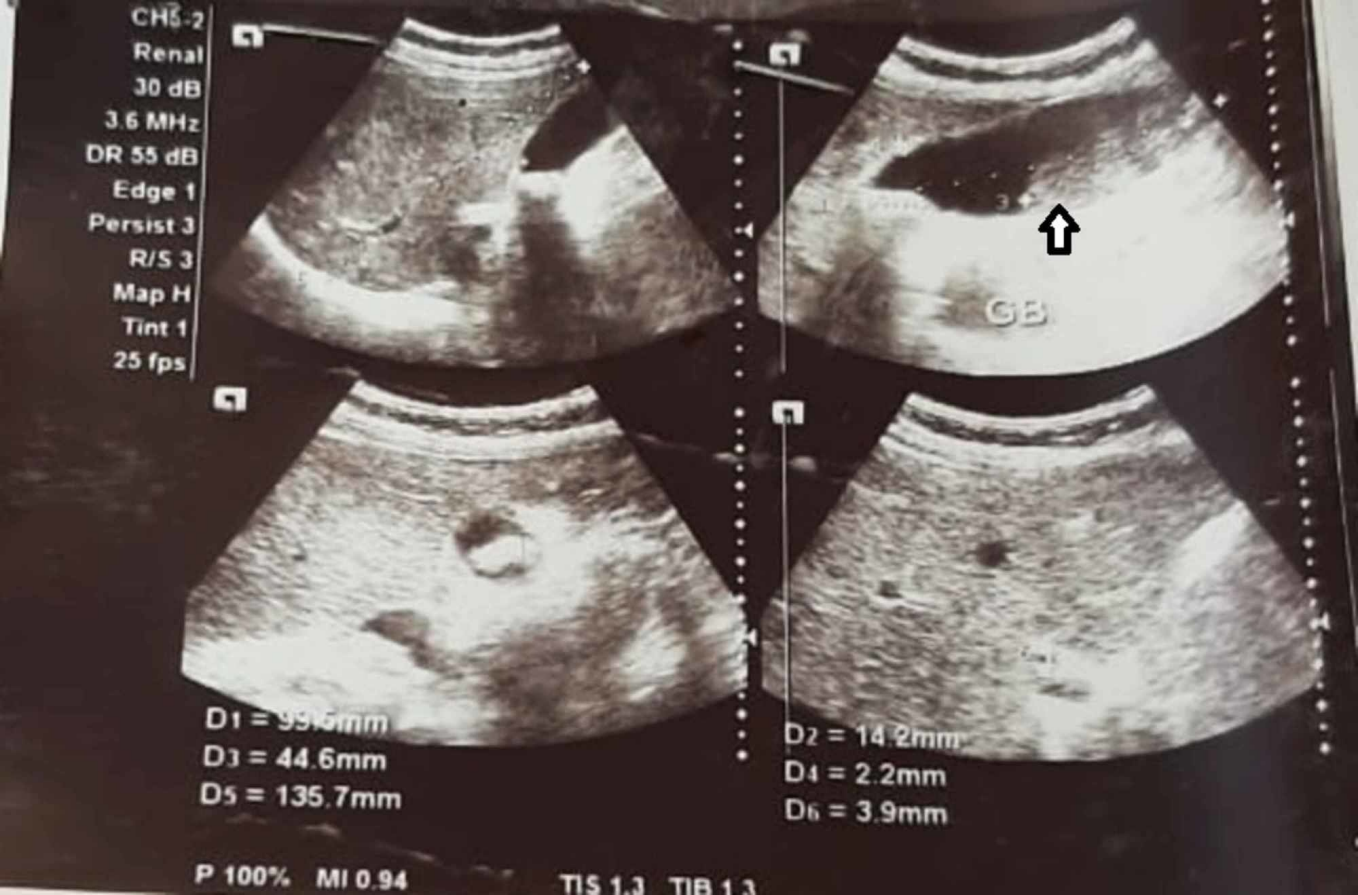
Ultrasound abdomen and pelvis with the arrowhead showing the distended gall bladder with multiple calculi with sludge

**Figure 2:**
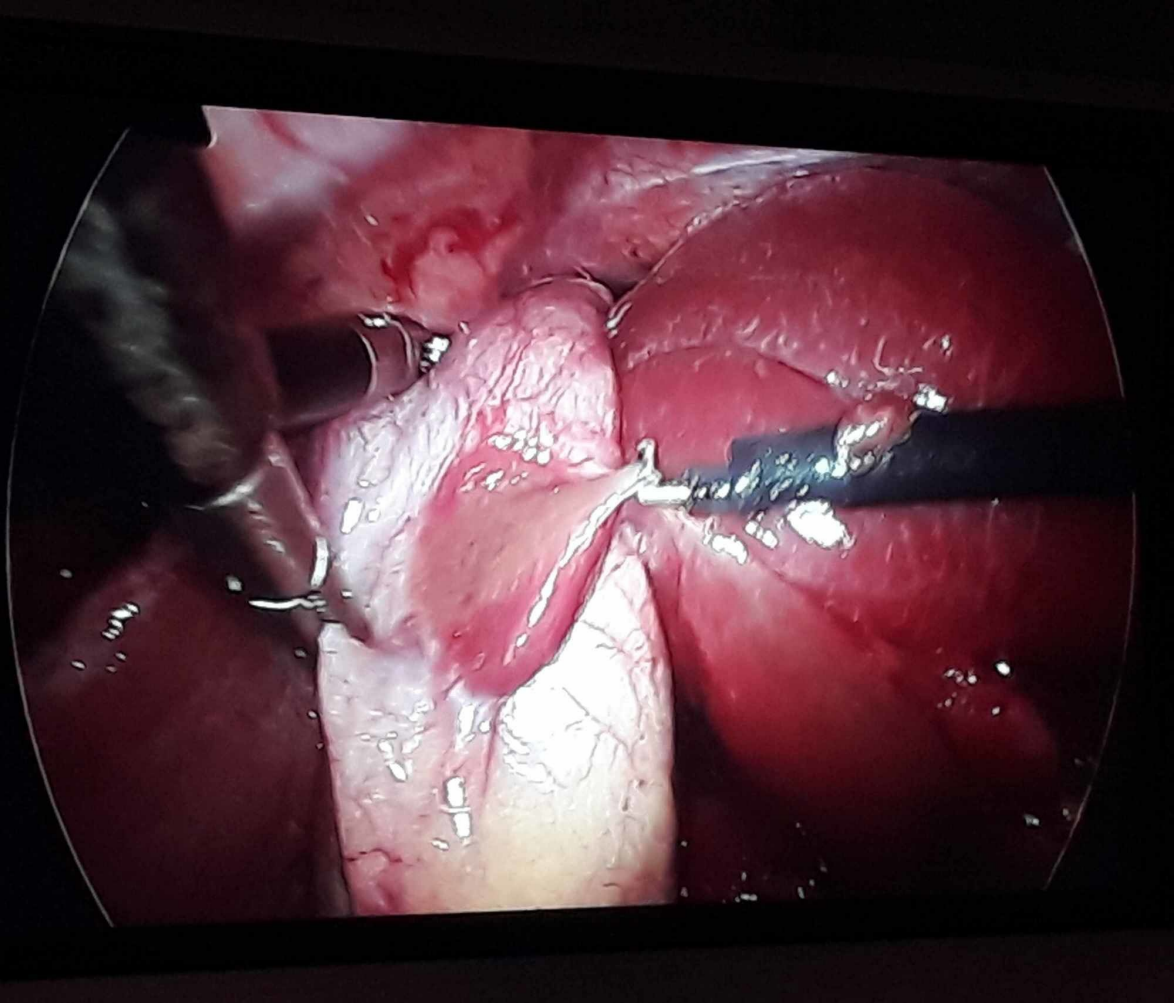
Ectopic liver-like tissue adherent to the anterior gall bladder wall connected to the left liver with a layer of serosa, which can be seen hooked by the hook dissector

**Figure 3:**
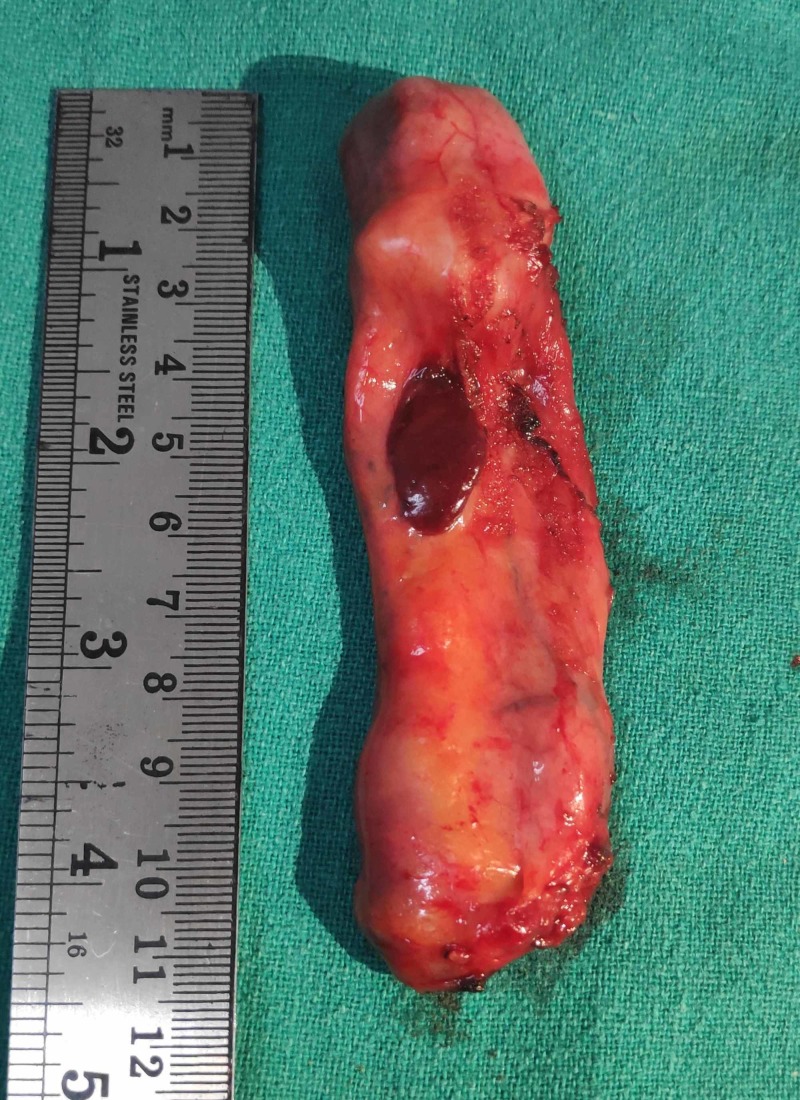
Distended gall bladder, approximately 11*4 cm with multiple calculi, along with ectopic liver-like tissue around 3*2 cm at the anterior aspect of the gall bladder

**Figure 4:**
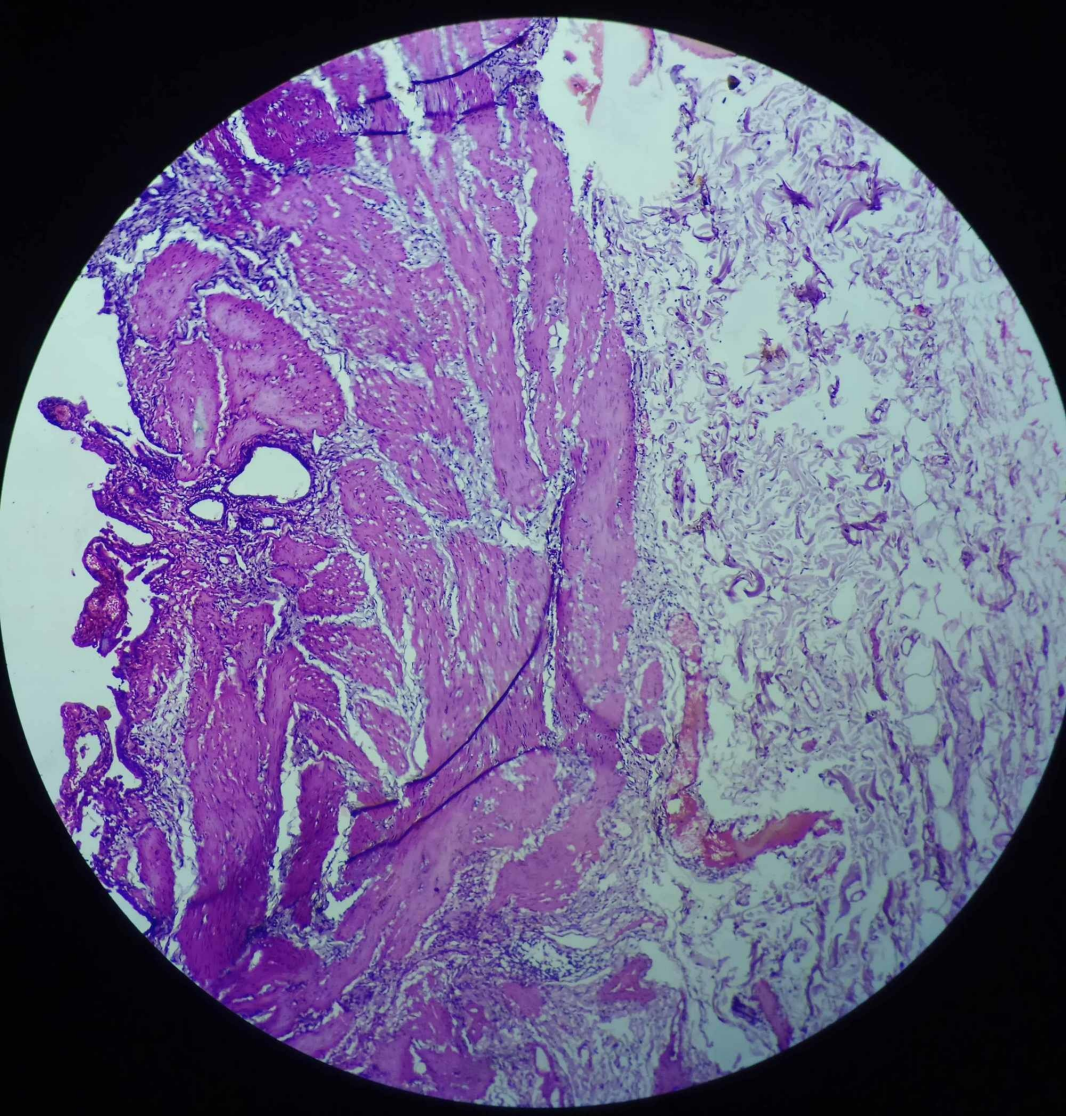
Section from gall bladder specimen showing lamina propria and submucosal fibrosis suggestive of chronic inflammatory changes

**Figure 5:**
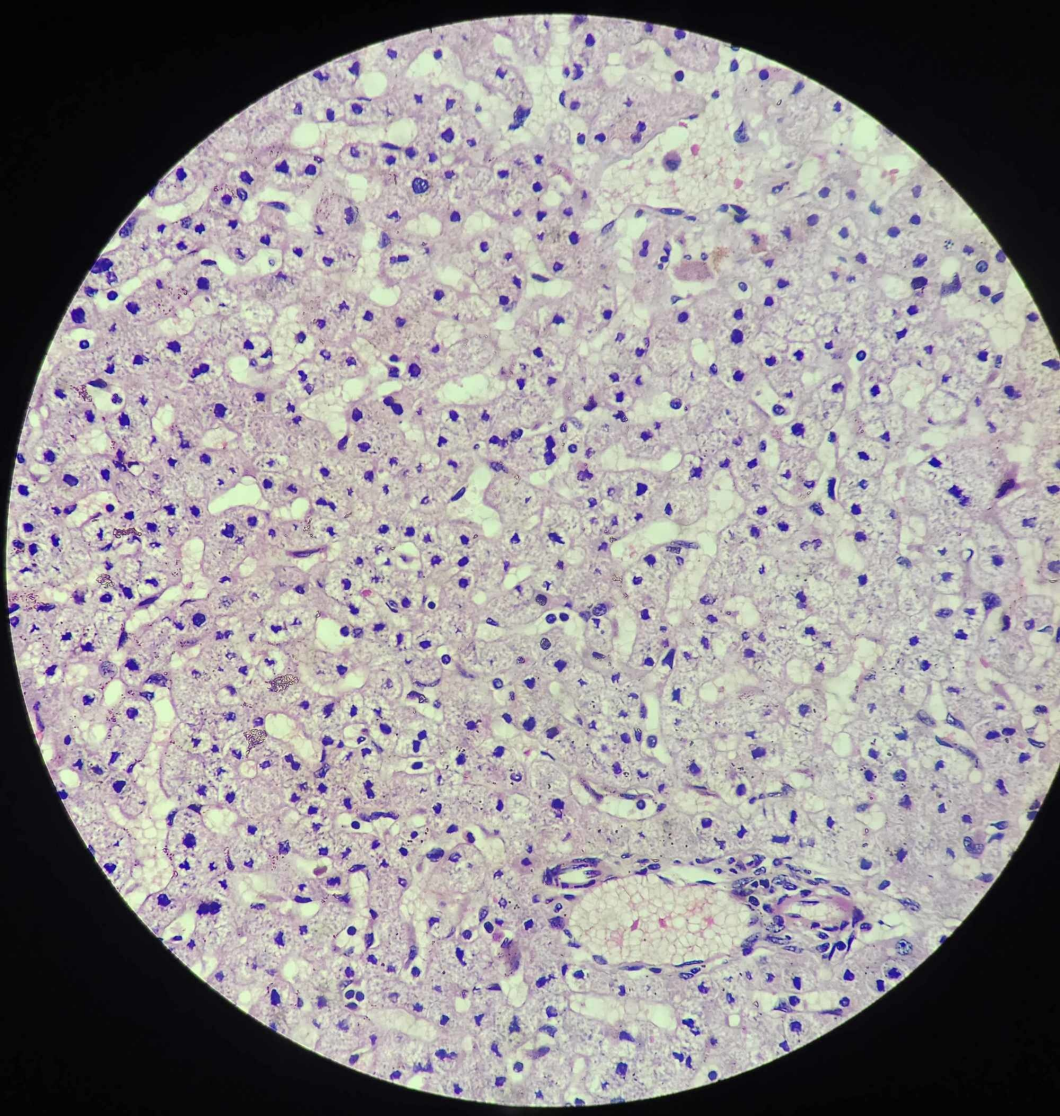
Representative section from attached tissue to the serosa shows normal hepatic tissue with capsule and hepatocytes (hematoxylin & eosin stain)

A 67-year-old lady presented with the complaint of pain in the right upper abdomen for three years, aggravated with fatty meals and relieved with medications. All laboratory parameters were within normal limits, with ultrasonography abdomen showing a distended gall bladder with calculi along with sludge (Figure 1). Her symptomatology and clinical examination were suggestive of symptomatic cholelithiasis. She underwent laparoscopic cholecystectomy and intraoperatively, her gall bladder was found to be distended and, to our surprise, a red-colored liver-like tissue was appreciated anterior to the gall bladder wall. This red-colored liver-like tissue was approximately 3 x 3 cm, connected to the left liver by a layer of serosa (Figure 2) The suspected ectopic liver tissue was removed along with the gall bladder and the specimen was sent for histopathology (Figure 3). The postoperative period remained uneventful and the patient was discharged home. Histopathology revealed liver tissue adhered by the layer of serosa to the gall bladder tissue. (Figure 4 and Figure 5).

## Discussion

Ectopic liver tissue (ELT) is one of the exceedingly rare conditions incidentally found both in the abdominal and thoracic cavity but clinically encounters limited to a few case reports [1]. By 2007, only 65 cases of ectopic liver, also termed hepatic choristomas, have been pointed out in the English literature [2]. It is thought to arise from aberrant migration of hepatic tissue that develops together with cystic structures from a foregut diverticulum and can be appreciated elsewhere intra-abdominally or intrathoracically [3]. Similarly, the close relation of the developing hepatic parenchyma cell cords to pars cystica may explain why the gall bladder could be embarked by the ectopic liver tissue [4].

ELT can rarely be diagnosed before surgical procedures or autopsies. It can be ignored easily by radiological techniques, with only a small number of cases been diagnosed preoperatively because it is often asymptomatic. Usually, they are found incidentally during surgery or while on post-mortem examinations and the reported prevalence is 0.24%-0.5%, with the gall bladder being the most common site [1].

Anatomically, ELT in the gall bladder derives its blood supply either from the vascular pedicle arising with or without its own vein from the liver parenchyma or from branches of the cystic artery and sometimes through vascular structures embedded within the mesentery lying adjacent to the liver parenchyma [3]. Surgically, it becomes important to delineate the blood supply because, often, the operating surgeon might encounter uncontrollable bleeding if the blood supply has been derived from liver parenchyma itself [5]. Complications that can be associated with ectopic liver are torsion, bleeding into the peritoneum, cirrhosis, and sometimes leading to malignant degeneration to hepatocellular carcinoma due to metabolic inactivity owing to less efficient vascular and biliary ductal systems, which sometimes might be confused for occult metastases from a primary hepatoma [1,6-7]. A study by Arakawa et al. has shown that one out of 42 ELT in the gall bladder was diagnosed as malignant [8]. Congenital anomalies like omphalocele, caudate lobe agenesis, biliary atresia, and bile duct cysts have been showed to have an association with ectopic liver [5].

Gall bladder-associated ELT is best managed by en bloc resection via laparoscopic cholecystectomy, which suffices if the biopsy comes out to be negative. However, as malignant degeneration chances still exist in about 3% of cases, some patients might need to undergo a second surgery for negative resection margin and regional lymphadenectomy [1,8-9].

## Conclusions

To conclude, though gall bladder-associated ELT is a rare anomaly, surgeons might encounter it during their surgical career, and as various reports in the literature quoted the chances of malignancy, proper histopathological evaluation and patient follow-up is mandatory. This case report is the first of its kind from this region, and we believe this highlights the importance of ELT in the gall bladder and possible interventions and complications that might be encountered, which need to be ascertained by every treating physician worldwide.

## References

[1] Martinez CA, de Resende HC Jr, Rodrigues MR, Sato DT, Brunialti CV, Palma RT (2013). Gallbladder-associated ectopic liver: a rare finding during a laparoscopic cholecystectomy. Int J Surg Case Rep.

[2] Pandit N, Sah R, Lacoul R (2019). Ectopic liver tissue on the gallbladder: a rare incidental finding. Indian J Surg.

[3] Bal A, Yilmaz S, Yavas BD (2015). A rare condition: ectopic liver tissue with its unique blood supply encountered during laparoscopic cholecystectomy. Int J Surg Case Rep.

[4] Catani M, De Milito R, Romagnoli F (2011). Ectopic liver nodules: a rare finding during cholecystectomy. G Chir.

[5] Mani VR, Farooq MS, Soni U, Kalabin A, Rajabalan AS, Ahmed L (2016). Case report of ectopic liver on gallbladder serosa with a brief review of the literature. Case Rep Surg.

[6] Asselah T, Condat B, Cazals-Hatem D (2001). Ectopic hepatocellular carcinoma arising in the left chest wall: a long-term follow-up. Eur J Gastroenterol Hepatol.

[7] Arslan Y, Altintoprak F, Serin KR, Kivilcim T, Yalkin O, Ozkan OV, Celebi F (2014). Rare entity: ectopic liver tissue in the wall of the gallbladder - a case report. World J Clin Cases.

[8] Arakawa M, Kimura Y, Sakata K, Kubo Y, Fukushima T, Okuda K (1999). Propensity of ectopic liver to hepatocarcinogenesis: case reports and a review of the literature. Hepatology.

[9] Koh CE, Hunt R (2007). Ectopic liver encountered during laparoscopic cholecystectomy. Asian J Surg.

